# Does the suddenness matter? Antidepressant use before and after a spouse dies suddenly or expectedly of stroke

**DOI:** 10.1177/14034948211042501

**Published:** 2021-10-05

**Authors:** Elina Einiö, Niina Metsä-Simola, Riina Peltonen, Pekka Martikainen

**Affiliations:** 1 Population Research Unit, Department of Social Research, Faculty of Social Sciences, University of Helsinki, Finland; 2 Laboratory of Population Health, Max Planck Institute for Demographic Research, Germany; 3 Department of Public Health Sciences, Stockholm University, Sweden

**Keywords:** Stroke, mental health, record linkage, depression, elderly

## Abstract

*Aims:* Changes in mental health at the time of widowhood may depend on the expectedness of spousal death, but scant evidence is available for spousal deaths attributable to stroke. *Methods:* Using register-linkage data for Finland, we assessed changes in antidepressant use before and after spousal death for those whose spouses died suddenly of stroke between 1998 and 2003 (*N*=1820) and for those whose spouses died expectedly of stroke, with prior hospitalisation for cerebrovascular disease (*N*=1636). We used both population-averaged logit models and individual fixed-effects linear probability models. The latter models control for unobserved time-invariant heterogeneity between the individuals. *Results:* Our study indicates that the suddenness of a spouse’s death from stroke plays a role in the well-being of the surviving spouse. Increases in antidepressant use appeared larger following widowhood for those whose spouses died suddenly of stroke relative to those whose spouses had a medical history of cerebrovascular disease. ***Conclusions:* The suddenness of a spouse’s death from stroke plays a role for the surviving spouse. The results suggest multifaceted timings of distress surrounding spousal death, depending on the suddenness of a spouse’s death from stroke.**

## Introduction

Risk factors for incident stroke and consequences of stroke for disability have been examined in previous studies [1], but less is known about mental health in families with members who die of stroke. The role of family members has been increasingly studied in relation to their potential contribution to prognosis and survival, given that survival after stroke is better for those who are married and for those who have offspring [2–4]. However, little evidence is available on changes in mental health for the surviving spouses before and after spousal deaths attributable to strokes, although stroke is globally one of the leading causes of death and adult disability [1].

The first aim of our study was to assess whether the sudden spousal death from stroke is worse for the surviving spouse’s mental health after the death than an expected death preceded by a medical history of cerebrovascular disease. Previous psychological literature has indicated that the experience of loss, threat or harm depends, among other factors, on the event predictability, controllability and perceived intent [5]. In our study, both types of spousal deaths from stroke – sudden and expected – are attributable to similar natural causes of death, without any intent to cause injury or death. Sudden and expected spousal deaths from stroke are psychologically different only in terms of their predictability. It has been suggested that the suddenness of a stressful life event, such as the death of a significant person, influences the way individuals appraise the event cognitively [5]. For instance, the sudden death might leave the survivor with less time to compensate for social and emotional losses and cause more health problems [6]. To our knowledge, however, no prior study has assessed whether the suddenness of a spouse’s death from stroke plays a role in the well-being of the surviving spouse, other things kept as constant as possible.

Our second aim was to assess whether an expected spousal death from stroke is worse for mental health prior to the death. There is evidence to suggest that a spousal death attributable to dementia is especially damaging for the surviving spouse during the years prior to widowhood, indicating that caregiving responsibilities are likely to affect the well-being of the surviving spouse [7]. Furthermore, an earlier study of life satisfaction showed larger decreases in life satisfaction prior to spousal death for older individuals, indicating larger anticipatory effects [8]. It is possible that older people mentally prepare for the loss prior to the death of a spouse, given that widowhood is more common at older ages [8,9]. However, no prior study has assessed whether the expectedness of a spouse’s death from a certain cause plays a role in the well-being of older individuals over the transition to widowhood.

In order to achieve our aims, we conducted a record-linkage study for Finland, using records for deaths and hospitalisations for the decedent spouses and antidepressant purchases for the surviving spouses surrounding widowhood. We used population-averaged logit models to analyse between-individual differences observed over the transition to widowhood. We also used individual fixed-effects linear probability models to analyse changes in antidepressant use in order to capture unobserved heterogeneity better between individuals.

## Methods

We carried out a record-linkage study based on a 14% sample of individuals aged ⩾40 years who resided in Finland on 31 December 1997. These individuals were linked with the members of their households, and all those included were followed for deaths until 31 December 2003 (permission number: TK-53-373-09). We restricted the data set to 3456 married individuals whose spouses died of stroke between 1998 and 2003. Of these widowed individuals, 53% experienced the spouse’s sudden death from stroke, and 47% the spouse’s more expected death from stroke. We used the International Statistical Classification of Diseases and Related Health Problems (ICD) to identify deaths and hospital diagnoses of stroke, using codes I60–I69 (ICD-10) and 430–434, 436–4376, 4378X–438 (ICD-9) for cerebrovascular diseases.

The death of a spouse was considered as sudden if no hospitalisations for cerebrovascular disease occurred prior to 30 days before the death and as expected if a spouse had been hospitalised for cerebrovascular diseases at least once in 1988–2003, and these hospitalisations occurred at least 30 days prior to the death. The 30-day criterion was used to include those with less than 30-day survival after stroke in the sudden-death category.

The Drug Prescription Register was used to form the outcome of antidepressant purchases, using Anatomical Therapeutic Chemical codes N06A for antidepressants and N06CA01 for antidepressants in combination with psycholeptics. The time to and since widowhood was divided into 12 six-month (183-day) observation periods around spousal deaths from stroke. The outcome was coded as 1 if purchases occurred during the observation period and 0 otherwise. The maximum follow-up time was 6.0 years, and the average follow-up time was 5.2 years. The follow-up time was right censored in the next period following the end of the follow-up time on 31 December 2003 or death, whichever came first.

We first employed population-averaged logit models to examine changes in the predicted probability of antidepressant purchases for three years before and three years after widowhood by the expectedness of spousal death. These results are displayed as six-month adjusted probabilities, estimated at the mean value of predictors. The model is adjusted for sex and time-varying indicators of age (continuous) and year (categorical). In Table II, the first two columns display the adjusted probabilities for the two widowed groups surrounding spousal death. The next column displays the difference between the groups. The final column displays the difference in changes in probabilities relative to their first observation period of 30–36 months before widowhood. The corresponding unadjusted probabilities are presented in Supplemental Table SI.

We then used individual fixed-effects linear probability models to estimate changes in antidepressant use within individuals. Individual fixed-effects models allow controlling for unobserved time-invariant characteristics of the individuals, such as their medical history, personality and genetic susceptibility to depressive symptoms that may otherwise induce estimation bias. These findings are presented as regression coefficients, which are multiplied by 100 so that they can be interpreted as percentage point changes relative to the 30–36 months before the spouse’s death (Table III, models 2–3). Had we used individual fixed-effects logit models, the pattern of findings would have been rather similar. These additional results are displayed in Supplemental Table SII.

## Results

The mean age at the time of the spouse’s death from stroke was 72.2 years. Of these individuals, 53% experienced the spouse’s sudden death from stroke, and 47% experienced the spouse’s expected death from stroke. The mean age at widowhood was 70.7 years for those whose spouses died suddenly of stroke and 73.8 years for those whose spouses died more expectedly of stroke (Table I).

**Table I.** Distribution of the study population and the proportion ever using antidepressants during the follow-up time for three years before and three years after the spouse’s death from stroke in 1998–2003, according to the suddenness of spousal death, Finland.

	Spouse dies expectedly of stroke (%)	Spouse dies suddenly of stroke (%)	Total: Spouse dies of stroke (%)	Total (*n*)	Using antidepressants (%)
Mean age at the spouse’s death (*SD*)	73.8 (9.1)	70.7 (10.9)	72.2 (10.2)		
Suddenness of the spouse’s death from stroke					
Yes	−	100.0	52.7	1820	26.1
No	100.0	−	47.3	1636	28.5
Sex					
Male	26.4	33.2	30.0	1036	21.9
Female	73.6	66.8	70.0	2420	29.5
Total	1636	1820	100.0	3456	27.2

*SD*: standard deviation.

The results from the population-averaged logit model show that the adjusted probability of antidepressant use was higher prior to widowhood for those whose spouses died expectedly of stroke compared to those whose spouses died suddenly of stroke (Table II). The difference was relatively modest for two to three years preceding widowhood but became larger and significant during the last year preceding a spouse’s death. Six to twelve months before widowhood, the adjusted probability of antidepressant use was 8.1% for those whose spouses died suddenly of stroke and 10.4% for those whose spouses had a medical history of hospitalisations for cerebrovascular disease. The group differences levelled off after their spouses died (13.8% sudden; 13.2% expected), given that the increase was larger when the spouses died suddenly. The results in the final column in Table II show that the probability of antidepressant use increased by approximately two percentage points more following widowhood for those whose spouses died suddenly rather than expectedly, relative to the levels observed at the beginning of the follow-up.

**Table II.** Trajectories of antidepressant use before and after spouses died expectedly or suddenly of stroke in 1998–2003, predicted probabilities, difference in probabilities and difference in changes in probabilities (Model 1: population-averaged logit model), Finland.

Months before/after spouse’s death	Spousal death expected	95% CI	Spousal death sudden	95% CI	Difference in predicted probability between groups	Difference in changes^a^ in predicted probability between groups
	Predicted prob.		Predicted prob.			
	(%)		(%)			
−30 to −36	10.35	8.61–12.09	9.01	7.36–10.65	−1.34	Ref.
−24 to −30	10.90	9.23–12.56	9.05	7.52–10.59	−1.84+	−0.50
−18 to −24	9.98	8.46–11.49	8.81	7.39–10.23	−1.17	0.17
−12 to −18	10.68	9.17–12.18	8.98	7.61–10.35	−1.69+	−0.35
−6 to −12	10.39	8.94–11.85	8.09	6.83–9.35	−2.31*	−0.97
−0 to −6	11.79	10.24–13.35	9.09	7.77–10.41	−2.70*	−1.36
0 to 6	13.23	11.55–14.92	13.82	12.20–15.44	0.59	1.93+
6 to 12	11.89	10.19–13.59	12.91	11.25–14.56	1.01	2.35*
12 to 18	10.46	8.75–12.17	11.97	10.26–13.69	1.52	2.86*
18 to 24	11.13	9.24–13.03	10.78	9.04–12.52	−0.35	0.99
24 to 30	10.41	8.41–12.40	10.21	8.38–12.04	−0.20	1.15
30 to 36	10.50	8.33–12.66	9.27	7.40–11.14	−1.23	0.12
Observations						36,184
People						3456

All predictors at their mean value for probability.

* *p*<0.05; +*p*<0.1.

a Changes relative to the period observed 30–36 months prior to spousal death.

CI: confidence interval.

The findings from fixed-effects linear probability models confirmed the pattern of findings, indicating that the suddenness of the spouse’s death was strongly associated with increases in antidepressant use following spousal death for those whose spouses died suddenly of stroke (Table III). Their six-month prevalence of antidepressant use was six percentage points higher shortly following spousal death relative to their first period observed three years prior to widowhood. The corresponding change for those who experienced the expected spousal death from stroke was less than two percentage points immediately following the spouse’s death. The use of antidepressants appeared to be on the decline for both groups upon the time since the spouse’s death. These further post-widowhood estimates lack statistical precision, however.

**Table III.** Trajectories of antidepressant use before and after spouses died expectedly or suddenly of stroke in 1998–2003, the coefficients and the predictions from individual fixed-effects linear probability models, Finland.

Months before/after spouse’s death	Fixed-effects linear probability models					
	Model 2			Model 3		
	Spousal death expected		Predicted probability	Spousal death sudden		Predicted probability
	Coefficient	95% CI	(%)	Coefficient	95% CI	(%)
−30 to −36	Ref.		12.15	Ref.		8.00
−24 to −30	0.36	−0.94 to 1.66	12.51	0.22	−0.94 to 1.38	8.22
−18 to −24	−0.69	−2.51 to 1.14	11.46	0.19	−1.76 to 2.14	8.19
−12 to −18	−0.18	−2.51 to 2.16	11.98	0.50	−2.12 to 3.12	8.50
−6 to −12	−0.70	−3.71 to 2.31	11.45	−0.21	−3.56 to 3.14	7.79
−0 to −6	0.56	−3.02 to 4.14	12.71	1.05	−3.02 to 5.13	9.06
0 to 6	1.89	−2.44 to 6.22	14.05	6.25*	1.35 to 11.15	14.26
6 to 12	0.18	−4.64 to 4.99	12.33	5.55+	−0.08 to 11.19	13.55
12 to 18	−1.73	−7.26 to 3.80	10.42	4.83	−1.55 to 11.22	12.83
18 to 24	−1.17	−7.34 to 4.99	10.98	3.77	−3.38 to 10.93	11.78
24 to 30	−2.32	−9.20 to 4.55	9.83	3.42	−4.49 to 11.32	11.42
30 to 36	−2.40	−9.83 to 5.04	9.76	2.61	−6.09 to 11.32	10.61
Observations	17,009			19,175		
People	1636			1820		

Coefficients are multiplied by 100 to obtain percentage-point change, relative to the first observation period. All predictors at their mean value for probability.

* *p*<0.05; +*p*<0.1.

## Discussion

The present study assessed whether the suddenness of a spouse’s death from stroke played a role in the mental well-being of the surviving spouse before and after spousal death. Our findings indicated that the probability of antidepressant use was higher before widowhood, in particular during the last marital year, when the spouse died expectedly rather than suddenly of stroke. The finding is of policy importance, given that a large proportion of individuals may survive their first stroke [10] and their spouses may become responsible for their care. It is possible that an elevated survival after stroke among the married [3] is accompanied by adverse mental-health consequences for their caregiving spouses. There is prior evidence to suggest that spousal deaths attributable to Alzheimer’s disease and other dementias – which are relatively expected and potentially burdensome for the caregiving spouses – are especially damaging for the survivors’ mental health years before widowhood [7]. Furthermore, an earlier study of life satisfaction showed larger decreases in life satisfaction prior to spousal death for older individuals, indicating larger anticipatory effects for older people. In addition, life satisfaction appeared lower prior to widowhood for those whose spouses had disabilities [8].

The findings from our fixed-effects models indicated that those whose spouses died suddenly rather than expectedly of stroke experienced large increases in antidepressant use shortly following the death of a spouse compared to the period observed three years prior to spousal death. Our findings cannot directly be compared to those of earlier studies because the mental-health consequences of the suddenness of spousal death from stroke have not been directly investigated before. Few studies have assessed whether the risk of mortality is differently elevated for those whose spouse died following a long-term illness or suddenly [6] or due to different causes of death [11]. For example, Smith and Zick [6] classified spousal deaths as expected when decedent spouses had medical conditions that began more than six months before their deaths based on death certificates, and they classified them as sudden when they died of sudden myocardial infarction or of suicide or car accident without long-term illness. Their findings indicated that younger widowers had higher mortality risk when their spouses died suddenly rather than expectedly compared to comparably aged married men [6]. It was suggested that sudden death might leave the survivor with less time to compensate for social and emotional losses and cause a severe sense of helplessness. However, suddenness was not associated with mortality increases among widows or older widowers [6]. Furthermore, the study did not focus specifically on spousal deaths from stroke.

Moreover, it is noteworthy that spousal death from suicide, violence or accident – sometimes categorised as sudden spousal death – is likely to affect the survivor’s well-being in many fundamental ways other than its suddenness alone. Krychiw et al. [12] summarised that the element common to suicides, homicides and fatal accidents that distinguishes them from other causes of death is the violent manner of the loss, which has a damaging effect on the psychological well-being of the bereaved. Empirical studies have shown that those who lost their spouse or parent to violent death had more severe grief symptoms compared to those who lost their significant other to non-violent death [12,13]. It is noteworthy that suicides are not always unexpected, in the sense that people might have had prior hospitalisations for suicide attempts before dying [14].

Empirical studies of the suddenness of spousal death other than violent death are seldom available. In a study of widowed people from the USA, Elwert and Christakis [11] classified certain cancers as quick cancers, including cancers of the head, neck, upper gastrointestinal tract, liver, central nervous system or pancreas and melanoma, lymphoma and leukaemia, based on cancer survival times at the time of the study. Their findings indicated that when a spouse died of quick cancer, the survivor’s mortality risk was not particularly elevated compared to when the deceased spouse died of other causes. The increase appeared relatively modest. They argued that this finding suggests that it might be the inevitability of the spouse’s death rather than the duration of the illness that buffers the survivor from some of the adverse effects of bereavement. However, this argument runs in contrast with that of Smith and Zick [6], who argued that the sudden death of a spouse could be particularly damaging because there is less time to foresee, adapt and compensate for the forthcoming loss.

Of course, expectedness of the death of a spouse based on the deceased spouse’s medical history or death certificate could be rather different from expectedness from the survivor’s subjective perspective. To measure subjective expectedness of the death of a spouse, Siflinger [15] used a question in an interview completed after the death of a spouse that asked the widowed the following: ‘Was the death expected at about the time it occurred, or was it unexpected?’ The study, which employed panel data for health measures, showed large increases in depressive symptoms at the time of the spouse’s death, particularly for those not expecting the death. Furthermore, Siflinger [15] assessed four distinct causes of spousal death: cancer, heart disease, respiratory disease and nutritional or digestive disease. She found that spousal death from cancer, heart disease and respiratory disease increased depressive symptoms at the time of widowhood compared to those observed prior to widowhood, and that unexpectedness had an additional adverse effect following spousal death from heart disease. This is in concordance with our finding that those whose spouses died unexpectedly rather than expectedly of stroke had larger increases in antidepressant use following the loss. However, the categorisation of causes of death (heart disease vs. stroke), the measure of expectedness (subjective ex post measure vs. objective ex ante measure) and the country (USA vs. Finland) were different from our study. On the contrary, an earlier American study of the consequences of the subjective expectedness of spousal death called into question the common belief that depressive symptoms among older people are more severe if the death is sudden [16]. The differences between the interpretations of the studies might relate to the fact that it is methodologically different to compare the levels of depression after the loss between two or three groups facing the loss than to assess changes in depressive symptoms following the loss compared to the levels observed before the loss among different groups [15,16].

The strengths of the current study are the high-quality register data of deaths and hospitalisations for cerebrovascular disease, linkage of the spouses, fixed-effects models and the continuous timescale for antidepressant purchases, divided into 12 six-month intervals surrounding widowhood. Individual fixed-effects models allowed controlling for unobserved time-invariant characteristics of the bereaved, such as their medical history, personality and genetic susceptibility to depressive symptoms that may otherwise induce estimation bias. We also distinguished between the expected and the sudden death of a spouse using prior hospitalisations for cerebrovascular disease. This is a unique feature of our study, given that the data included information on the dates of hospitalisation for stroke and of deaths from stroke. However, survey designs could still capture the subjective feelings of surprise better than register-linkage studies such as ours.

Our study has other limitations. One limitation is that the use of antidepressants as the outcome is an indirect measure of depressive disorders [7]. It has been shown that antidepressants have psychiatric indications other than depression. So, only around half of users meet the criteria for major depressive disorder [17,18], and in Finland, a quarter of antidepressant users have no psychiatric diagnosis [18]. Non-psychiatric use is unrelated to education but is somewhat more common in older age groups [18]. The mean age at the spouse’s death was >72 years in our register-linkage study, and it cannot be ruled out that some people might have used antidepressants for non-psychiatric purposes. In addition to non-psychiatric use of antidepressants, there is also a large proportion of individuals with a psychiatric disorder not receiving pharmacological treatment [19]. There is prior evidence to suggest that only a third of individuals with depression receive treatment in Finland [20], and the use of pharmacological treatment is even lower [21–23]. Evidence from Denmark also indicates that there is a social gradient in undertreatment in that individuals in the lower social group are less likely to receive treatment for their depression [24]. In addition, another Danish study comparing survey- and register-based measures of depression showed that high self-reported depression scores were more strongly associated with excess mortality than were medication-based measures of depression [25]. These results imply that register-based depression indicators do not capture all individuals with depression. On the other hand, register-linkage studies allow for minimising the loss to follow-up after the spouse’s death, which is important, given that the bereaved with severe grief symptoms could be at risk of non-response in surveys. In our study, however, the magnitude of changes in antidepressant use obtained from the fixed-effects models is subject to statistical uncertainty, given the small number of individuals whose spouses died of stroke. Unfortunately, the follow-up data for the spouse’s death were available to us only from 1998 to 2003.

Another limitation of our study is that register-linkage studies do not allow us to consider changes in health behaviour, including alcohol use or physical exercise, that could be related to widowhood and mental health. Assessing the contribution of health behaviour in explaining the difference between those whose spouses died suddenly or expectedly offers promise as a direction for future study. In our study, we studied the suddenness of spousal death by including those with less than 30-day survival after stroke in the sudden-death category. It is, however, worth noting that it is still an issue what cut-off point would be the best to assess the suddenness of spousal death, and this should be dealt with in future studies. Future research could also explore the dynamics of loss further by examining how the well-being of widowed individuals is affected by their decedent spouses’ survival times.

## Conclusions

This is the first study to assess whether the suddenness of a spouse’s death from stroke plays a role in the mental well-being of the surviving spouse before and after spousal death. Our study indicates that the sudden death of a spouse from stroke may be worse for mental health shortly after spousal death than the expected death from stroke, although it may spare individuals from the stresses of caregiving. The results suggest multifaceted timings of distress surrounding widowhood, depending on the suddenness of a spouse’s death from stroke.

## Supplemental Material

sj-docx-1-sjp-10.1177_14034948211042501 – Supplemental material for Does the suddenness matter? Antidepressant use before and after a spouse dies suddenly or expectedly of stroke Click here for additional data file. Supplemental material, sj-docx-1-sjp-10.1177_14034948211042501 for Does the suddenness matter? Antidepressant use before and after a spouse dies suddenly or expectedly of stroke by Elina Einiö, Niina Metsä-Simola, Riina Peltonen and Pekka Martikainen in Scandinavian Journal of Public Health

sj-docx-2-sjp-10.1177_14034948211042501 – Supplemental material for Does the suddenness matter? Antidepressant use before and after a spouse dies suddenly or expectedly of stroke Click here for additional data file. Supplemental material, sj-docx-2-sjp-10.1177_14034948211042501 for Does the suddenness matter? Antidepressant use before and after a spouse dies suddenly or expectedly of stroke by Elina Einiö, Niina Metsä-Simola, Riina Peltonen and Pekka Martikainen in Scandinavian Journal of Public Health
