# Supplementary material for: Does the suddenness matter? Antidepressant use before and after a spouse dies suddenly or expectedly of stroke
Source: Scand J Public Health. 2021 Oct 5;51(1):75–81. doi: 10.1177/14034948211042501 (PMC9900187; doi:10.1177/14034948211042501)
Supplement: sj-docx-1-sjp-10.1177_14034948211042501 – Supplemental material for Does the suddenness matter? Antidepressant use before and after a spouse dies suddenly or expectedly of stroke [file sj-docx-1-sjp-10.1177_14034948211042501.docx]

**Supplementary Table 1.** Unadjusted 6-month probability of antidepressant use for three years before and three years after the spouse's death from stroke in 1998–2003, according to the suddenness of spousal death, Finland, N = 3456

| Months before/after spouse's death | Spousal death expected | Spousal death sudden |
| --- | --- | --- |
|  | Unadjusted prob. | Unadjsuted prob. |
|  | (%) | (%) |
| -30-36 | 9.2 | 7.2 |
| -24-30 | 10.2 | 7.6 |
| -18-24 | 9.8 | 7.9 |
| -12-18 | 11.1 | 8.5 |
| -6-12 | 11.4 | 8.1 |
| -0-6 | 13.1 | 9.3 |
| 0-6 | 15.0 | 14.6 |
| 6-12 | 14.0 | 14.0 |
| 12-18 | 12.7 | 13.4 |
| 18-24 | 13.8 | 12.4 |
| 24-30 | 13.2 | 12.0 |
| 30-36 | 13.6 | 11.2 |
