# Supplementary material for: Does the suddenness matter? Antidepressant use before and after a spouse dies suddenly or expectedly of stroke
Source: Scand J Public Health. 2021 Oct 5;51(1):75–81. doi: 10.1177/14034948211042501 (PMC9900187; doi:10.1177/14034948211042501)
Supplement: sj-docx-2-sjp-10.1177_14034948211042501 – Supplemental material for Does the suddenness matter? Antidepressant use before and after a spouse dies suddenly or expectedly of stroke [file sj-docx-2-sjp-10.1177_14034948211042501.docx]

**Supplementary Table 2.** Trajectories of antidepressant use before and after spouses died expectedly or suddenly of stroke in 1998–2003, the odds ratios from individual fixed-effects logit models, Finland

|  | Fixed-effects logit models | | |  |
| --- | --- | --- | --- | --- |
|  | Model (4) |  | Model (5) |  |
| Months before/after spouse's death | Spousal death expected |  | Spousal death sudden |  |
|  | Odds ratio | 95% CI | Odds ratio | 95% CI |
|  |  |  |  |  |
| -30-36 | 1.00 | (ref.) | 1.00 | (ref.) |
| -24-30 | 1.13 | [0.74 - 1.73] | 1.08 | [0.71 - 1.63] |
| -18-24 | 0.86 | [0.49 - 1.54] | 1.07 | [0.62 - 1.83] |
| -12-18 | 1.00 | [0.47 - 2.12] | 1.19 | [0.60 - 2.37] |
| -6-12 | 0.89 | [0.35 - 2.31] | 0.98 | [0.41 - 2.33] |
| -0-6 | 1.17 | [0.37 - 3.69] | 1.35 | [0.48 - 3.79] |
| 0-6 | 1.52 | [0.39 - 5.90] | 4.10* | [1.22 - 13.80] |
| 6-12 | 1.07 | [0.22 - 5.13] | 3.56+ | [0.88 - 14.43] |
| 12-18 | 0.72 | [0.12 - 4.29] | 3.05 | [0.62 - 15.03] |
| 18-24 | 0.82 | [0.11 - 6.00] | 2.45 | [0.41 - 14.59] |
| 24-30 | 0.65 | [0.07 - 6.03] | 2.21 | [0.31 - 16.03] |
| 30-36 | 0.63 | [0.06 - 7.15] | 1.82 | [0.21 - 15.88] |
| Observations | 4319 |  | 4708 |  |
| Persons | 412 |  | 438 |  |
| * p<0.05, + p<0.1 | |  |  |  |
| Note: All predictors at their mean value for probability. | | | |  |
| Fixed-effects logit models require within-individual variation in the outcome. | | | | |
